# Endoscopic Surveillance in Idiopathic Achalasia

**DOI:** 10.7759/cureus.17436

**Published:** 2021-08-25

**Authors:** Olive Ochuba, Sheila W Ruo, Tasnim Alkayyali, Jasmine K Sandhu, Ahsan Waqar, Ashish Jain, Christine Joseph, Kosha Srivastava, Sujan Poudel

**Affiliations:** 1 Internal Medicine, California Institute of Behavioral Neurosciences & Psychology, Fairfield, USA; 2 General Surgery Research, California Institute of Behavioral Neurosciences & Psychology, Fairfield, USA; 3 Internal Medicine, Marmara University, Istanbul, TUR; 4 Pathology, California Institute of Behavioral Neurosciences & Psychology, Fairfield, USA; 5 Obstetrics and Gynecology, California Institute of Behavioral Neurosciences & Psychology, Fairfield, USA; 6 Family Medicine, California Institute of Behavioral Neurosciences & Psychology, Fairfield, USA; 7 Urology & Obstetrics and Gynecology, California Institute of Behavioral Neurosciences & Psychology, Fairfield, USA; 8 Neurology, California Institute of Behavioral Neurosciences & Psychology, Fairfield, USA; 9 Psychiatry and Behavioral Sciences, California Institute of Behavioral Neurosciences & Psychology, Fairfield, USA; 10 Division of Research & Academic Affairs, Larkin Community Hospital, South Miami, USA

**Keywords:** achalasia, cancer, esophageal squamous cell carcinoma, esophageal adenocarcinoma, screening, surveillance, esophageal dysmotility

## Abstract

Idiopathic achalasia is a rare esophageal dysmotility disorder of unknown etiology with only palliative treatment available. Many studies have established a significantly increased risk of esophageal cancer in patients with achalasia. However, current guidelines advise against routine surveillance due to low absolute risk and a paucity of high-quality evidence and cost-effectiveness assessments. This review aims to assess the need for routine endoscopic surveillance in achalasia based on a growing body of literature calling in support of it, mainly due to the increased risk of esophageal cancer. We searched PubMed and Google Scholar electronic databases for articles within the last 10 years using the keywords 'achalasia', 'cancer,' 'neoplasms,' 'screening,' and 'surveillance.' After excluding pseudoachalasia/secondary achalasia, other esophageal dysmotility disorders, and associations with malignancies outside the esophagus, we selected 31 articles for this review. Through these articles, we identified areas of focus for ongoing and future research that may result in significant risk reduction of complications, including esophageal cancer and beyond.

## Introduction and background

Idiopathic achalasia is a progressive esophageal motility disorder characterized by esophageal aperistalsis and failure of the lower esophageal sphincter (LES) to relax spontaneously [1,2]. With an incidence of approximately 1 in 100,000 and a prevalence of 10 in 100,000, it is classified as a rare disease [3]. Although the exact etiology remains undetermined, the leading opinion favors the likely virus-triggered autoimmune destruction of the inhibitory neurons of the esophageal myenteric plexus in a genetically predisposed individual [2,4-6].

The main symptoms include insidious onset of dysphagia to both solids and liquids, regurgitation of undigested food and saliva, and retrosternal chest pain with meals. Some patients also report consequent weight loss [5,7]. While no definitive cure exists, highly effective palliative management offers good temporary symptom control for most patients [8,9]. For most, surgical treatment options are considered first-line focusing on the LES and offering dilation or a reduction in its pressure [2]. These options include graded pneumatic dilatation, (laparoscopic) myotomy, also known as Heller myotomy (HM), and peroral endoscopic myotomy (POEM) as a minimally invasive option. Esophagectomy is largely reserved for end-stage disease [2,5,10]. Nonsurgical management is often an adjunct to surgical procedures or the mainstay in poor surgical candidates. They include botulinum toxin injection into the LES, proton pump inhibitors for acid reflux control, calcium channel blockers, phosphodiesterase inhibitors, and nitrates for esophageal spasm [1,2]. The medical treatments have significantly lower efficacy levels than other management options, with some guidelines such as the International Society for Diseases of the Esophagus (ISDE) recommending against their use for symptomatic relief due to a lack of convincing evidence [2]. Generally, the nonsurgical management options provide short-term benefits, and the surgical treatments offer relatively longer-term symptom control. However, recurrent periods of inadequate control are common and result in greater morbidity and poorer outcomes, the most feared being the development of esophageal cancer.

The association between idiopathic achalasia and increased risk of esophageal cancer is well documented; however, there are currently no standardized screening recommendations [11]. This review aims to assess the need for routine surveillance in patients with achalasia based on a growing body of literature calling in support of it. Most of the current guidelines cannot support routine cancer surveillance in achalasia due to insufficient evidence demonstrating cost-effectiveness and a relatively low incidence of esophageal cancer. The topic remains controversial with varying practices. This review aims to consider the risk of esophageal malignancy in achalasia and other poorer outcomes that can be prevented with endoscopic surveillance.

We searched the PubMed and Google Scholar electronic databases with keywords 'achalasia', 'cancer,' 'neoplasms,' 'screening,' and 'surveillance' from the last 10 years with 3012 unique resulting articles. Through title and abstract review of the resulting articles, we selected 60 articles by excluding those relating to pseudoachalasia/secondary achalasia, other esophageal dysmotility disorders, and malignancies outside the esophagus. After the full-text screening, we selected 31 articles for this review consisting of two meta-analyses, 12 reviews, three guidelines, and 14 observational studies.

## Review

Esophageal cancer in achalasia

Esophageal cancer is the seventh most common cancer in men and the 13th most common in women [12]. Achalasia is associated with an overall increased risk of esophageal cancer, with a significantly higher risk of squamous cell carcinoma (SCC) than adenocarcinoma (AC). This increased risk of esophageal cancer is higher in men than women [13]. The difference between the sexes is not yet fully understood but would most logically follow from the already known increased risk of esophageal cancer seen in men compared to women, as achalasia affects both sexes equally [14]. A recent meta-analysis by Tustumi et al. reviewed 40 studies that included a total of 11,978 achalasia patients and reported the incidence of esophageal SCC as 312.4 cases per 100,000 patient-years at risk compared with a 4.3 in 100,000 patient-years at risk in the general population [15]. The study reported the incidence of esophageal AC as 21.23 cases per 100,000 patient-years at risk in achalasia patients [15]. The prevalence of esophageal cancer was 28 cases in 1,000 achalasia patients. The prevalence of esophageal SCC is 26 in 1,000 achalasia cases compared with four in 1,000 for esophageal AC [15].

Squamous cell carcinoma of the esophagus in achalasia

**Figure 1:**
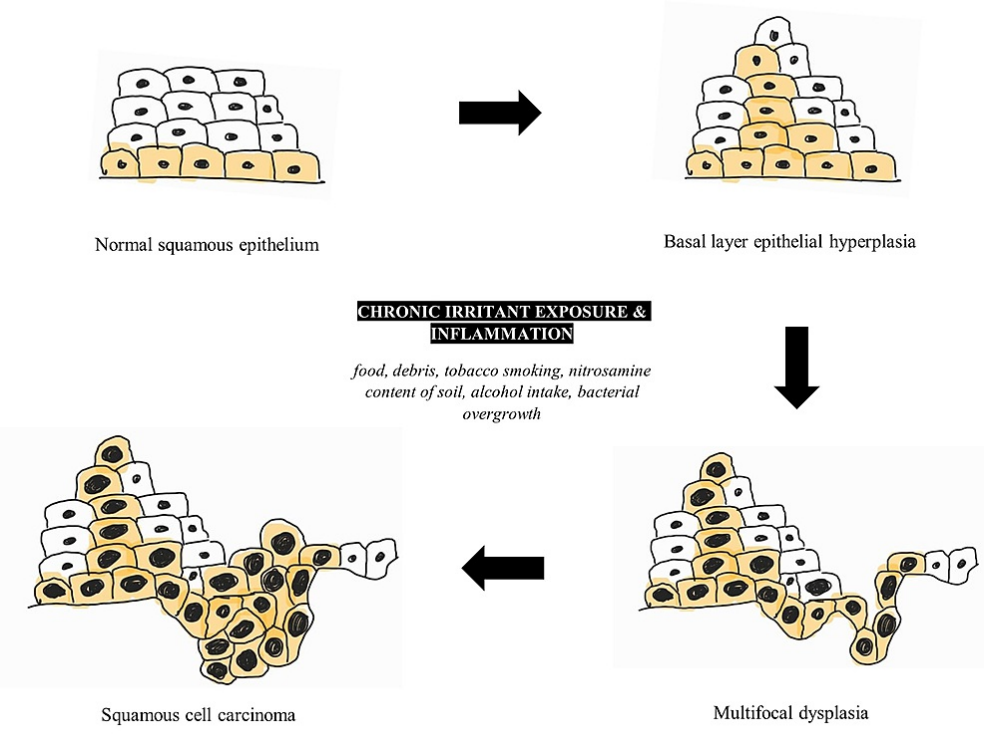
The epithelial hyperplasia to esophageal squamous cell carcinoma sequence An original schematic diagram of the progression from esophageal normal squamous epithelium to esophageal squamous cell carcinoma, highlighting the main risk factors.

Esophageal SCC more often affects the upper two-thirds of the esophagus, and esophageal AC occurs in the lower third, near the LES and the gastroesophageal junction [5]. The significant risk factors reported were cigarette smoking, alcohol, burns from ingestion of drinks at high temperatures, diets rich in processed and red meat, salty foods, nitrosamines, human papillomavirus infection, and achalasia [4,11,16,17]. The epithelial hyperplasia to esophageal SCC sequence is shown in Figure 1. Negative correlations, and therefore protective factors, highlighted were diets rich in vegetables and weight maintenance within recommended body mass indexes [17]. These risk factors cause repeated inflammation to the pseudostratified squamous epithelial lining of the esophagus resulting in the transformation to carcinoma. The review article by Tustumi et al. (2021) noted that achalasia is associated with a 16-28% increase in the risk of SCC that is primarily due to the increased stasis of irritants and carcinogens caused by the aperistaltic esophagus, thereby increasing contact time with the epithelial lining [4]. It is important to note that achalasia affects the entire length of the esophagus as peristalsis is severely impaired. Peristalsis is required for the successful passage of the food bolus along the entire length of the esophagus. Therefore, in achalasia, there is a chronically increased contact time of the food/irritant with the epithelium than would typically occur.

Longstanding irritation and inflammation of the mucosal lining results in chronic hyperplastic esophagitis progressing to multifocal dysplasia, culminating in an increased risk of SCC development [10,18]. A study using immunohistochemistry staining cluster of differentiation 3 (CD3), cluster of differentiation 20 (CD20), proliferation marker Ki-67, and tumor suppressor gene p53 examined the histopathological changes visible on the middle and lower esophageal endoscopic biopsies from 22 patients with achalasia and 17 controls. The study found significantly higher levels of inflammation, a greater number of CD3-positive than CD20-positive lymphocytes, and a higher Ki-67 proliferation index in patients with achalasia. The tumor suppressor gene p53, although statistically non-significant in this study, was noted in only achalasia patients [19]. Other studies with larger cohorts have shown statistically significant associations with the p53 gene in borderline dysplastic and carcinoma in situ lesions in achalasia patients [18,20]. The study concludes that such findings may be valuable in evaluating endoscopic biopsies from patients with achalasia allowing early detection of malignant transformation, which is particularly important in esophageal carcinoma where the prognosis is usually poor due to late detection [17].

Adenocarcinoma of the esophagus in achalasia

**Figure 2:**
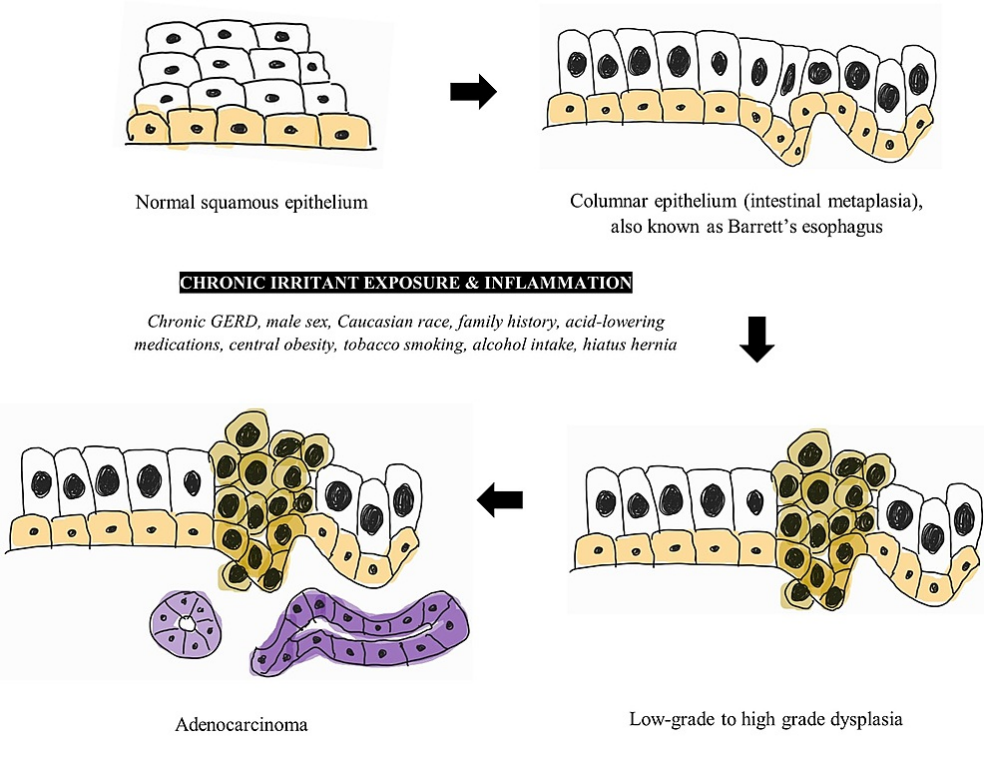
The Barrett’s esophagus to esophageal adenocarcinoma sequence An original schematic diagram of the progression from esophageal normal squamous epithelium to esophageal adenocarcinoma via Barrett’s metaplasia, highlighting the main risk factors.

Esophageal AC is recognized mainly as the endpoint in a sequence transformation in the lower esophagus. Under well-known risk factors, the normal lower esophageal stratified squamous epithelium is transformed into columnar epithelium, notably a premalignant metaplastic lesion termed Barrett’s esophagus (BE). This change is the response of the epithelium to chronic exposure to gastric acid and is, therefore, most characterized as a complication of longstanding gastroesophageal reflux disease (GERD) [5]. BE may then progress from low-grade to high-grade dysplasia and then to AC. The BE to AC sequence is shown in Figure 2. The prevalence of BE is estimated between 1.3% and 1.6%, from Italian and Swedish population studies [21,22]. The annual risk of BE transforming to AC is 0.12% to 0.5% [23]. Although esophageal SCC is more common overall, AC of the esophagus is more prevalent in the developed world, and a stark increase in incidence in Western nations over the last 30 years has been recorded [11,24]. The prevalence of esophageal SCC is higher in the developing world, particularly in South-Eastern and Central Asia [11]. The reasons for the differing prevalences are not fully known, but trends in obesity and tobacco smoking in these areas may provide some useful correlations [11]. Risk factors for esophageal AC include chronic GERD, male sex, Caucasian race, positive family history, chronic use of acid-lowering medications, central obesity, tobacco smoking, alcohol, and the presence of a hiatus hernia [5].

Achalasia treated with a myotomy without anti-reflux manipulation (such as a partial fundoplication) of the gastroesophageal junction is associated with increased GERD [1]. For this reason, the European Society of Neurogastroenterology and Motility and United European Gastroenterology (ESNM/UEG) recommended follow-up endoscopy to screen for GERD in patients who have undergone myotomy without anti-reflux manipulation [1]. Although GERD is a known risk factor for esophageal AC, and achalasia patients are at increased risk of GERD, the ESNM/UEG suggest against routine screening for dysplasia and cancer [1]. However, the ESNM/UEG advises maintaining a low threshold for endoscopic assessment in recurrent symptoms and longstanding achalasia [1]. The post-myotomy prevalence of esophageal AC in achalasia patients is 7 in 1000 compared with an overall prevalence of 4 in 1,000 in those without myotomy performed [5]. An even lower incidence in esophageal AC was reported in those who underwent a partial fundoplication in addition to the myotomy [5]. 

Endoscopic surveillance in achalasia

Despite established associations between esophageal cancer and achalasia and recent studies estimating this significantly increased risk and the need for routine screening, most current guidelines cannot support screening due to a lack of evidence. Malignancy surveillance and follow-up in achalasia remain controversial, with practices varying widely amongst clinicians due to no consensus on screening.

The guidelines on the management of achalasia published in 2020 by the American Society of Gastrointestinal Endoscopy (ASGE) report insufficient evidence to support routine screening in this population. However, they suggest that surveillance endoscopy may benefit patients who have undergone a POEM due to the increased risk of reflux, but recognized that more evidence is needed to support a stronger recommendation [25]. The American College of Gastroenterology (ACG) 2020 guidelines agree with the ASGE concerning the lack of sufficient evidence to recommend routine screening [26]. The lack of high-quality studies investigating this specific issue (often with small sample sizes and lead-time and length-time bias), and the low overall absolute risk and incidence of esophageal cancer, make any recommendation for routine surveillance difficult [26]. The guidelines refer to a Swedish study from 1995 that found 406 surveillance endoscopies were needed to detect one cancer in the first year after diagnosis of achalasia [27]. However, this should not be used to estimate yield in a cancer surveillance program as risk increases with achalasia progression. Furthermore, an endoscopic evaluation is performed as part of the diagnostic workup in achalasia for the primary purpose of excluding malignancy. Esophageal malignancy diagnosed within one year of an idiopathic achalasia diagnosis is uncommon. Most of the recent studies evaluating the risk of esophageal cancer in achalasia patients exclude cancer diagnosis within the first 12 months from diagnosis for this reason. With this exclusion, the average time from achalasia diagnosis to esophageal cancer diagnosis ranges from 5 to 15 years [1,2].

Interestingly, the ACG remark upon the possible benefits and growing interest in surveillance endoscopy beyond malignancy surveillance. For example, a three-yearly assessment in those with an achalasia history of greater than 10-15 years assessing for risk of progression to megaesophagus may be beneficial [26]. Arguably, a similarly aged cohort of patients has been described to be at increased risk of malignancy and would benefit most from surveillance endoscopy. There is also great difficulty in assessing the true length of disease history in the patient with achalasia. This is not solely due to delayed patient presentation resulting from insidious symptom onset but also a consequence of a recognized diagnostic delay mainly due to a misdiagnosis of GERD alone [28] and poor clinician recognition of typical symptomatology [29]. Diagnostic delay is currently reported as two years from patient presentation to a confirmed diagnosis [29]. Although this is an improvement from previous years, better education and awareness are needed [29]. Future investigations seeking to assess such benefits may be better designed to consider the time from diagnosis as a standard measure.

Also referred to by the ACG guidelines is a long-term prospective cohort study between 1975 and 2006 where 448 achalasia patients were followed post graded pneumatic dilatation for an average of 9.6 years with endoscopic surveillance evaluation and biopsies [30]. Fifteen patients developed esophageal cancer at a mean age of 71 years after an average of 11 years from diagnosis and an average of 24 years from symptom onset. In this study, five of the 15 patients received potentially curative treatment, an outcome that most likely would not have resulted had no surveillance been undertaken given the poor five-year survival of less than 15% in esophageal cancer [24]. The study concluded a low absolute risk of development of esophageal cancer, despite a significantly increased risk in achalasia patients.

The ESNM/UEG recommends a low threshold for endoscopic evaluation in achalasia patients with recurrent symptoms and longstanding disease [1]. Due to the nature of symptoms in achalasia, reliance upon the patient presenting with new or different symptoms, or even the ability to distinguish a difference if indeed one exists, would be inappropriate as an indication for malignancy screening. Typically, symptoms from a malignant process in the esophagus correlate with advanced achalasia and a poor prognosis. This recommendation from the ESNM/UEG would have greater utility in assessing for GERD rather than malignancy (largely dependent on the specific symptoms) but again draws attention to the high suspicion for a malignant disease that must be held in the management of the patient with achalasia.

In a recent study, 530 patients with achalasia were followed for a median of 50.5 months (inducing a greater than 10-year follow-up in 78 patients). Results showed that six patients (1.2%) developed esophageal cancer, reporting an incidence of 219.8 in 100,000 person-years. All six patients with esophageal cancer were detected early, and potentially curative resections were performed [9]. Not mentioned in the study, but may be of interest, is the incidence of high-grade dysplasia noted on endoscopy, which can be managed to reduce risk further. Many recent studies have joined a support for considering routine endoscopic surveillance in achalasia patients as a tool for early detection and therefore improved prognosis in esophageal cancer and high-grade dysplasia [1,9,13]. A 2020 review article in the *Annals of Esophagus* (AOE) journal noting the guidelines of the ASGE and ACG brought attention to the differences in management recommendations for conditions with shared risk factors and commonly seen conditions in achalasia such as BE, where surveillance is recommended [31]. This discrepancy is also seen in the ASGE’s recommendation for surveillance in post-POEM achalasia patients due to increased reflux, increasing the risk of AC, despite the much higher risk for SCC present from the diagnosis of achalasia alone. The review concluded that there might be benefits in the surveillance of patients with histories longer than 10 years, particularly in the presence of other risk factors such as male gender, alcohol intake, and tobacco smoking [31]. The value of routine screening in this group is gradually being considered. The European Society of Gastrointestinal Endoscopy (ESGE) released a statement last year recommending the use of endoscopic cancer screening for esophageal cancer in high-risk groups for SCC and AC [24]. Those at high risk for SCC include a history of head or neck cancer, achalasia, or prior caustic injury. At high risk for Barrett's esophagus-associated AC are those with chronic GERD (greater than five years) and risk factors such as age of at least 50 years, white race, male sex, obesity, and a first-degree relative with BE or esophageal AC [24]. No specific surveillance schedule is proposed in the ESGE recommendations, and surveillance strategies across Europe continue to vary.

Limitations

Despite gaps in literature determining the exact etiology of achalasia, strong risk factors and associations useful in managing and preventing serious complications have been identified. Uncertainties surrounding cancer surveillance policies result from a lack of random controlled trials and cost-effectiveness assessments of such a screening program. Ethical issues that may be difficult to overcome when designing trials for malignancy screening are likely to be a barrier, particularly concerning a malignancy with a longstanding poor prognosis. As a result of this, coupled with low prevalence rates, most of the studies are retrospective, of relatively small sample size, and an amalgamation of various endoscopic surveillance intervals. Additionally, many studies fail to highlight the value of early detection of high-grade dysplasia (for which effective, potentially curative therapies, and intensive surveillance strategies exist), focusing solely or mainly on carcinoma detection.

## Conclusions

A strong, established association between achalasia and the development of esophageal cancer exists, with recent literature calling for routine endoscopic cancer surveillance to be considered as an individual patient risk, particularly in men, is significantly high. However, most current guidelines cannot recommend this mainly due to insufficient, high-quality evidence available. More studies need to be performed to objectively assess the cost-effectiveness of screening as this is lacking in the current literature. Small studies focusing on the association between esophageal cancer and achalasia have highlighted a group (5 to 15 years from diagnosis) where biopsy results reported high-grade dysplasia and carcinoma. A greater proportion of those with esophageal cancer and high-grade dysplasia in these studies received potentially curative treatment due to early detection within the trials. Current guidelines note that there may be a role for routine endoscopic surveillance in specific groups of achalasia patients, such as in the assessment of GERD post-POEM or assessing risk in longstanding disease for the progression megaesophagus. Interestingly, the population of these two subsets essentially encompasses that of the group identified as high risk for developing esophageal cancer. Therefore, there seems to be value in paying particular attention to this subset. Future studies compounding the various benefits for surveillance endoscopy in this group, without a sole focus on early detection of malignancy, may obtain results of greater significance. This would allow patients to benefit from earlier detection of both benign and malignant complications, thereby improving chances of better outcomes.
